# Whole-genome sequence data of *Bacillus tropicus* strain MHES12 isolated from the rhizosphere of tomato in a drought-prone ecosystem in Bangladesh

**DOI:** 10.1016/j.dib.2026.113123

**Published:** 2026-08-05

**Authors:** Md. Nazmul Hannan, Md. Nayeem Hossain, Rifat Islam, Sabbir Khan, Kamrun Nahar, GKM Mustafizur Rahman, Habibul Bari Shozib, Abul Hossain Molla, M. Nazmul Hoque, Md. Manjurul Haque

**Affiliations:** a Department of Environmental Science, Faculty of Agriculture, Gazipur Agricultural University, Gazipur 1706, Bangladesh; b Molecular Biology and Bioinformatics Laboratory, Department of Gynecology, Obstetrics and Reproductive Health, Faculty of Veterinary Medicine and Animal Science, Gazipur Agricultural University, Gazipur 1706, Bangladesh; c Biotechnology Division, Bangladesh Agricultural Research Institute, Gazipur 1701, Bangladesh; d Department of Soil Science, Faculty of Agriculture, Gazipur Agricultural University, Gazipur 1706, Bangladesh; e Grain Quality and Nutrition Division, Bangladesh Rice Research Institute, Gazipur 1701, Bangladesh

**Keywords:** Rhizosphere bacteria, Comparative genomics, Biosynthetic gene clusters, Secondary metabolites, Sustainable agriculture, Draft genome

## Abstract

We report the whole-genome sequence data of *Bacillus tropicus* strain MHES12 isolated from the rhizosphere of tomato plants grown in a drought-prone ecosystem in Rajshahi, Bangladesh (24.44,067° N, 88.40,943° E). Genomic DNA was extracted using the GeneJET Genomic DNA Purification Kit, quality-checked by NanoDrop 2000 and Qubit 4.0 Fluorometer (Thermo Fisher Scientific) and sequenced on the Illumina MiSeq platform (2 × 150 bp) using the Nextera DNA Flex Library Preparation Kit. Raw reads were quality-assessed with FastQC v0.11.3, trimmed with Trimmomatic v0.39 (Q30 = 94% R1, 79% R2 raw; 96% and 88% after trimming), and de novo assembled with SPAdes v4.2.0, yielding a draft genome of 5569,334 bp across 40 contigs at 63× total read coverage (52.9× after trimming), with a GC content of 35.1%, N50 value of 838,108 bp and N90 value of 124,199 bp. CheckM2 (Neural-Network specific model) reported 100% completeness and 0.05% contamination. Taxonomic placement was confirmed by FastANI (96.98% to *B. tropicus* N24 type strain), skani (97.21%), NCBI ANI (96.90%) and TYGS dDDH (d4 = 72.5%), all above the 95% ANI and 70% dDDH species-delimitation thresholds. An additional publicly available complete *Bacillus tropicus* genome (EMB20, GCA_023159405.1; PRJNA742863) from India shares 99.12% FastANI with MHES12, further supporting the species assignment. A maximum-likelihood core-genome phylogeny built from 1119 single-copy core genes across 21 Bacillus genomes places MHES12 in the *B. tropicus* clade with 100% SH-aLRT/ultrafast-bootstrap support. Genome annotation using the NCBI Prokaryotic Genome Annotation Pipeline (PGAP) v6.11 predicted a total of 5829 genes, comprising 5707 coding sequences, 24 rRNA genes, 93 tRNA genes, 5 ncRNA genes, and 200 pseudogenes. antiSMASH v8.0 identified 10 biosynthetic gene clusters, including siderophore-associated clusters with petrobactin- and bacillibactin-like features, both of which were independently confirmed against reference operons in the Virulence Factor Database (VFDB), as well as ribosomally synthesized and post-translationally modified peptide (RiPP) clusters, a terpene cluster, and a beta-lactone synthetase cluster. As these biosynthetic cluster classes are commonly distributed among members of the *B. cereus* group, their identification is presented as a genome-based reference inventory rather than evidence of functional activity or pathogenicity. RAST subsystem analysis, cross-validated using eggNOG-mapper and Prokka, identified putative genomic determinants associated with osmotic and oxidative stress responses, as well as arsenic, chromium, copper, cadmiu*m/z*inc/cobalt, manganese, and iron homeostasis. These findings represent predicted genomic potential and should not be interpreted as evidence of phenotypic expression or experimentally verified resistance or stress tolerance. PathogenFinder2 predicted a low probability of human pathogenicity (score = 0.3912), below the 0.5 threshold. However, ABRicate analysis against the Virulence Factor Database (VFDB) identified the *B. cereus* group enterotoxin operons *nheA/B/C, hblA/C/D*, and *cytK*, which are widely distributed among environmental members of the *B. cereus* group. Nevertheless, the in silico detection of these genes alone does not establish a pathogenic phenotype, and their functional expression and potential pathogenic significance would require experimental validation. The annotated genome assembly (GenBank: JBXWLV000000000; version JBXWLV010000000) and raw sequencing reads (SRA: SRR36889416; BioProject: PRJNA1405269; BioSample: SAMN54739323) are publicly available for reuse in comparative genomics and *B. cereus* group taxonomy.

Specifications Table

**Table:** 

Subject	Biology
Specific subject area	Plant-associated bacteria; whole-genome sequencing; biosynthetic genomics; comparative taxonomy
Type of data	Table Figure Raw sequencing reads (FASTQ) Analyzed: genome assembly (FASTA), genome annotation (GFF/GenBank)
Data collection	Genomic DNA extracted using GeneJET Genomic DNA Purification Kit; quality and concentration verified with NanoDrop 2000 and Qubit 4.0 Fluorometer (Thermo Fisher Scientific). Library prepared using Nextera DNA Flex Kit (Illumina; 1 ng input DNA). Sequencing performed on Illumina MiSeq. Strain isolated from tomato rhizosphere by serial dilution and plating on yeast-extract peptone agar at 28 °C; identity confirmed by VITEK 2 Compact (biomérieux) and *16S rRNA* gene PCR. Sequencing coverage: 63.1× raw / 52.9× post-trim (SPAdes-reported: 29×); assembly with SPAdes v4.2.0; annotation with NCBI PGAP v6.11.
Data source location	Location: Rajshahi, Bangladesh Coordinates: 24.44,067° N, 88.40,943° E
Data accessibility	Repository name: NCBI GenBankBioProject: PRJNA1405269BioSample: SAMN54739323Assembly Accessions: • MHES12: GCA_057321065.1 Direct URL:https://www.ncbi.nlm.nih.gov/bioproject/PRJNA1405269
Related research article	None

## 1 Value of the Data

• The draft genome sequence of *B. tropicus* MHES12 provides an additional reference genome for the *B. tropicus* lineage from a South Asian agroecosystem.
• Phylogenetic analysis of MHES12 revealed its close evolutionary relationship with related *Bacillus* species.
• The 10 biosynthetic gene clusters (BGCs) identified (with > 90.0% identity) provide a starting point for comparative studies of iron-acquisition systems in the *B. tropicus* lineage.
• The genome harbours putative, genome-predicted determinants (genomic potential) associated with osmotic adaptation, oxidative stress defence, and homeostasis of arsenic, chromium, copper, cadmiu*m/z*inc/cobalt and manganese; these annotations require phenotypic confirmation.
• Four putative azoreductase genes (*azoR*) and 10 nitroreductase-family genes are also predicted. This gives researchers a candidate gene list for designing targeted expression or knockout studies of genome-predicted stress-tolerance and bioremediation-relevant traits (which remain to be experimentally verified), without needing to repeat genome mining.
• Publicly deposited raw reads (SRA: SRR36889416) and annotated genome assembly (GenBank: JBXWLV000000000) ensure full data reusability, reproducibility, and accessibility for future functional, comparative, and applied genomic studies involving drought-adapted plant-associated bacteria.

## 2 Background

*B. tropicus* is an endospore-forming, plant-associated bacterium increasingly recognised for its plant growth-promoting traits, biocontrol activity, and tolerance to environmental stressors [1,2]. Members of the genus *Bacillus* are widely utilised in sustainable agriculture as biofertilizers and biocontrol agents [[3], [4], [5]]. Despite this relevance, publicly available genomic data on native *B. tropicus* strains recovered from drought-prone agroecosystems in South Asia remain limited, constraining comparative and functional genomic studies in the region. To address this gap, we sequenced the genome of *B. tropicus* strain MHES12 isolated from the rhizosphere of tomato plants cultivated under drought conditions in Rajshahi, Bangladesh (24.44,067° N, 88.40,943° E). This data article is related to a companion genome report for *Priestia megaterium* MHES4 [3], thereby adding value to an expanding genomic dataset of plant-associated *Bacillus*-group bacteria from Bangladesh that may inform future development of locally adapted biofertilizer candidates.

## 3 Data Description

**Fig. 1:**
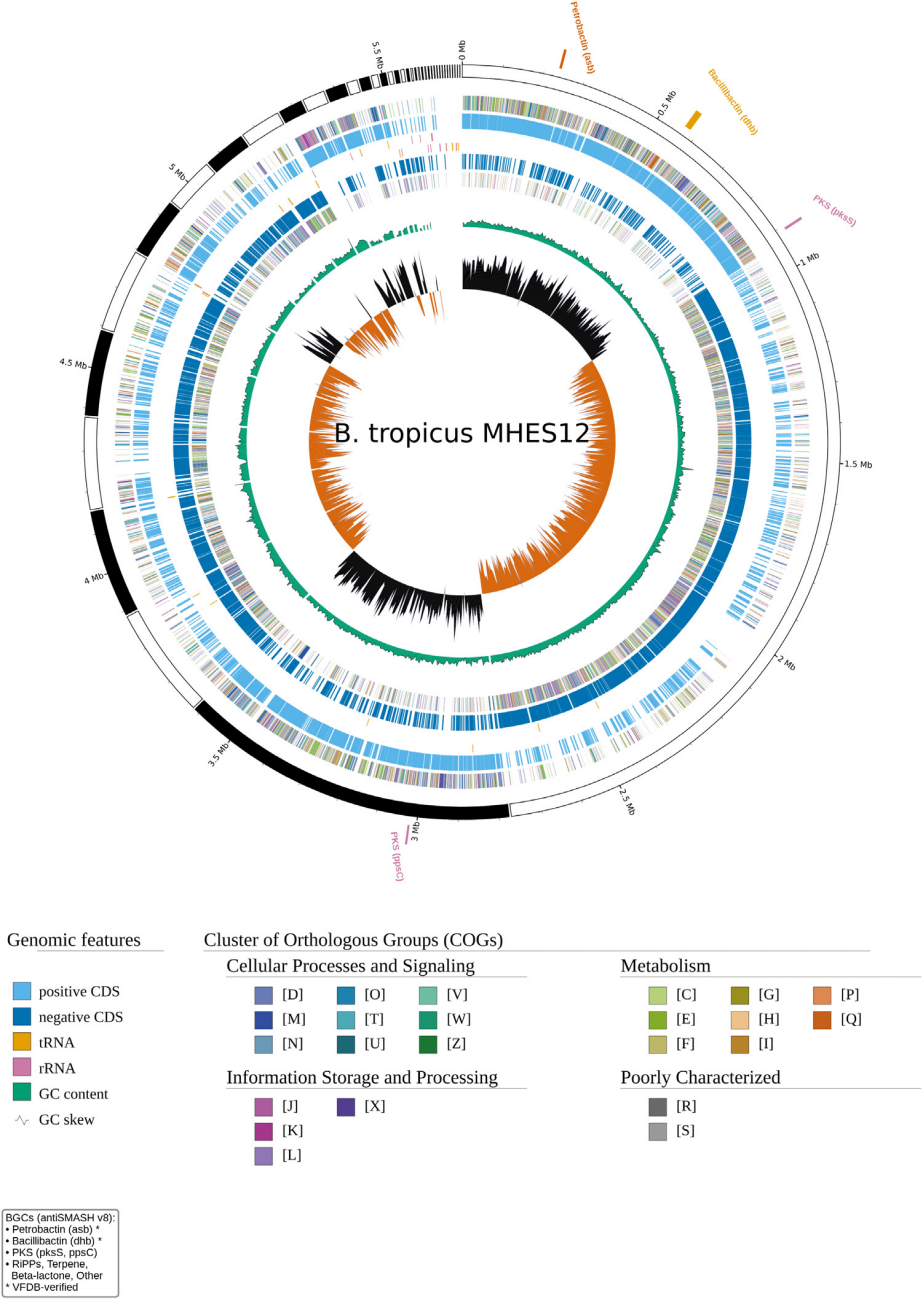
Circular genome map of *B. tropicus* MHES12 generated using GenoVi. The outermost ring overlays the 10 antiSMASH v8-predicted biosynthetic gene clusters at their resolved genomic positions, with the two VFDB-verified siderophore clusters (petrobactin/asb and bacillibactin/dhb) shown in distinct colours. Inner rings represent (i) positive-strand coding sequences (CDS; light blue), (ii) negative-strand CDS (dark blue), (iii) transfer RNA genes (orange), (iv) ribosomal RNA genes (yellow), (v) COG functional category assignments, (vi) GC content (green), and (vii) GC skew (orange, positive; black, negative). The genome spans 5.57 Mb across 40 contigs with a GC content of 35.1%.

The genomic characteristics of *B. tropicus* MHES12 are summarised in Table 1. The genome was sequenced using the Illumina MiSeq platform (2 × 150 bp), yielding 618,952 raw read pairs (1237,904 reads; 351.4 Mbp total; Q30 = 94% R1 / 79% R2, GC = 35.36% R1 / 35.67% R2). After adapter and quality trimming (Trimmomatic v0.39; LEADING:3, TRAILING:3, SLIDINGWINDOW:4:20, MINLEN:50) 547,936 read pairs (1095,872 reads; 88.5%; 294.9 Mbp; Q30 = 96% R1 / 88% R2) were retained, giving 63.1× raw and 52.9× post-trim total-bp coverage relative to the assembled genome size. De novo assembly with SPAdes v4.2.0 produced 40 contigs totalling 5569,334 bp, N50 = 838,108 bp, N90 = 124,199 bp, largest contig 2775,627 bp, GC = 35.1% (Fig. 1, Table 1). CheckM2 v1.0.2 (Neural-Network specific model, GTDB Rr220) reported 100.0% completeness and 0.05% contamination. Genome annotation by the NCBI Prokaryotic Genome Annotation Pipeline (PGAP) v6.11 predicted 5829 genes comprising 5707 coding sequences, 24 ribosomal RNA genes, 93 transfer RNA genes, 5 non-coding RNA genes and 200 pseudogenes. Full per-file read QC, assembly, ANI and dDDH metrics are provided in Table S1.

**Table 1:** Genomic features of *B. tropicus* MHES12 isolated from tomato rhizosphere in Bangladesh.

**Genomic Feature**	***B. tropicus*****MHES12**
GenBank accession no.	JBXWLV000000000
Genome version	JBXWLV010000000
BioProject accession no.	PRJNA1405269
BioSample accession no.	SAMN54739323
SRA accession no.	SRR36889416
Raw read pairs (Illumina MiSeq)	618,952 (1237,904 reads total)
Raw total bases (bp)	351,447,015
Q30% raw reads (R1 / R2)	94 / 79
GC% of raw reads (R1 / R2)	35.36 / 35.67
Duplication rate raw (R1 / R2%)	26.55 / 19.88
Trimmed read pairs (after Trimmomatic v0.39)	547,936 (1095,872 reads total; 88.5% retained)
Q30% trimmed reads (R1 / R2)	96 / 88
Duplication rate trimmed (R1 / R2, %)	25.80 / 21.52
Sequencing platform	Illumina MiSeq
Assembled genome size (bp)	5569,334
GC content (%)	35.1
Genome completeness (%)	100.0 (CheckM2 v1.0.2, Neural-Network specific model); 99.41 (CheckM v1.2.5)
Genome contamination (%)	0.05 (CheckM2, Neural-Network specific model)
Genome coverage (×)	63.1 (raw reads) / 52.9 (post-trim reads) / 29 (SPAdes-reported)
N₅₀ value (bp)	838,108
N₉₀ value (bp)	124,199
Largest contig (bp)	2775,627
L50 / L90	2 / 9
Number of contigs	40
Genes (total)	5829
Coding sequences (CDS)	5707
Pseudogenes	200
Ribosomal RNAs (rRNAs)	24
Transfer RNAs (tRNAs)	93
Non-coding RNAs (ncRNAs)	5
Biosynthetic gene clusters	10
CRISPR arrays	2 (evidence level 1)
PathogenFinder 2 score	0.3912 (predicted low probability of human pathogenicity)
Enterotoxin operons (ABRicate vs VFDB)	nheA/B/C, hblA/C/D, cytK present (95–99% identity); cereulide (ces) and anthrax (cya/lef/pagA) absent

Note. PathogenFinder2 score interpretation: 0.8–1.0, high likelihood of human pathogenicity; 0.5–0.8, moderate likelihood; < 0.5, low likelihood (more likely low probability of human pathogenicity).

Secondary metabolite e.g.*,* BGCs were predicted by antiSMASH v8.0, identifying 10 BGCs including siderophore clusters (petrobactin- and bacillibactin-like, both independently confirmed by ABRicate against the virulence factor database with the *asbA*-F and *dhbA*-F operons at 100% coverage), RiPPs, a terpene cluster and a beta-lactone synthetase (Fig. 1, **Table S2**). These cluster classes: siderophore, RiPP, terpene and beta-lactone are conserved components of the *B. cereus* group and are commonly recovered from *B. tropicus* and related taxa; the catalogue is therefore reported as a genome-based reference inventory that adds comparative value for the lineage rather than as evidence of novel chemistry. Two putative CRISPR arrays (evidence level 1) were detected by CRISPRCasFinder and no plasmid replicons were identified. PathogenFinder2 predicted a low probability of human pathogenicity (score = 0.3912; below the 0.5 threshold). ABRicate against the virulence factor database detected the *B. cereus* group enterotoxin operons nheA/B/C, hblA/C/D and cytK, characteristic of the group. These enterotoxin operons are widely distributed among environmental *B. cereus* group isolates, and their detection in-silico does not by itself indicate a pathogenic phenotype; cytotoxicity would require experimental verification (see Limitations). Anthrax toxin (cya/lef/pagA) and cereulide (ces) genes were absent (Tables S3–S4). ABRicate against VFDB additionally detected alo (anthrolysin), inhA (immune inhibitor A), ilsA (iron-regulated surface protein), and Type VII secretion components (essC/esxB); the full toxin and virulence-factor panel is provided in Table S4.

Functional subsystem analysis via RAST assigned coding sequences across 333 subsystems, cross-checked against Prokka and eggNOG-mapper annotations. The annotation recovered putative, genome-predicted determinants (genomic potential only) spanning four functional categories: osmotic adaptation (e.g., proline biosynthesis and glycine-betaine/choline transport), oxidative-stress defence (e.g., superoxide dismutases and catalase), homeostasis of arsenic, chromium, copper, cadmiu*m/z*inc/cobalt, manganese and iron (including the petrobactin *asbA*-F and bacillibactin *dhbA*-F iron-acquisition operons), and candidate xenobiotic-reduction enzymes (four FMN-dependent azoreductases [azoR] and ten nitroreductase-family proteins, with ring-cleaving mono- and dioxygenases [6]). Notably, dedicated mercury- (merA/merR), nickel- (nikABCDE) and lead-specific (pbrA) determinants were not identified. The complete locus-tagged inventory of these determinants, with annotation source for each hit, is provided in Tables S5–S7. These are annotation-based predictions only; physiological verification of osmotic/oxidative-stress tolerance, metal tolerance, azo-dye degradation and plant-growth promotion is beyond the scope of the present genomic report and will require targeted phenotypic assays (see Limitations).

**Fig. 2:**
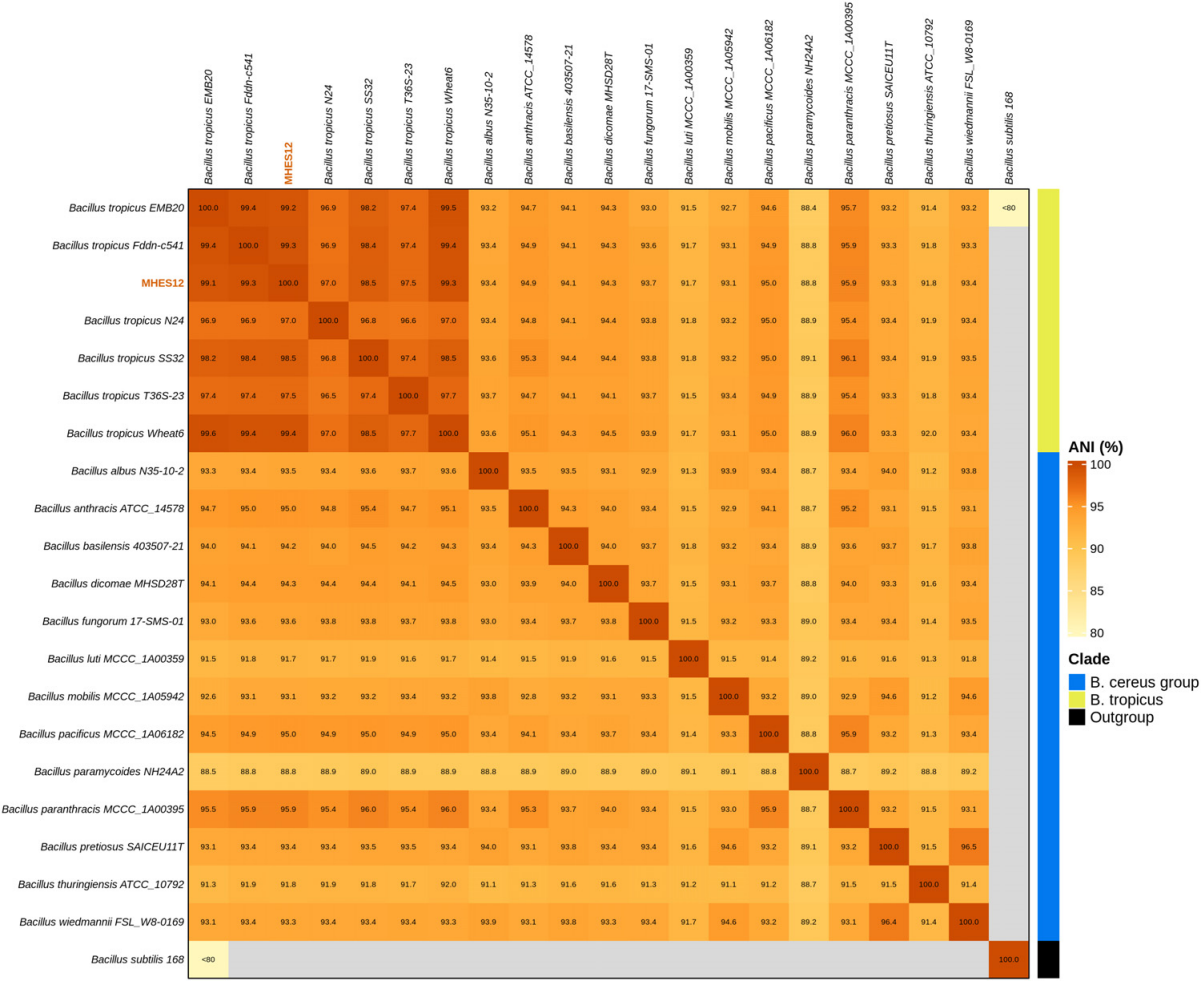
Whole-genome average nucleotide identity (ANI) heatmap among 21 Bacillus genomes. Pairwise FastANI values are shown; the colour scale ranges from 80% to 100% identity. MHES12 (bold) clusters with *B. tropicus* N24, the four in-house *B. tropicus* drafts (T36S-23, SS32, Wheat6, Fddn-c541), and the publicly available *B. tropicus* EMB20 at ANI values above 95%, clearly separated from other *B. cereus* group species. *B. subtilis* 168 serves as the outgroup.

**Fig. 3:**
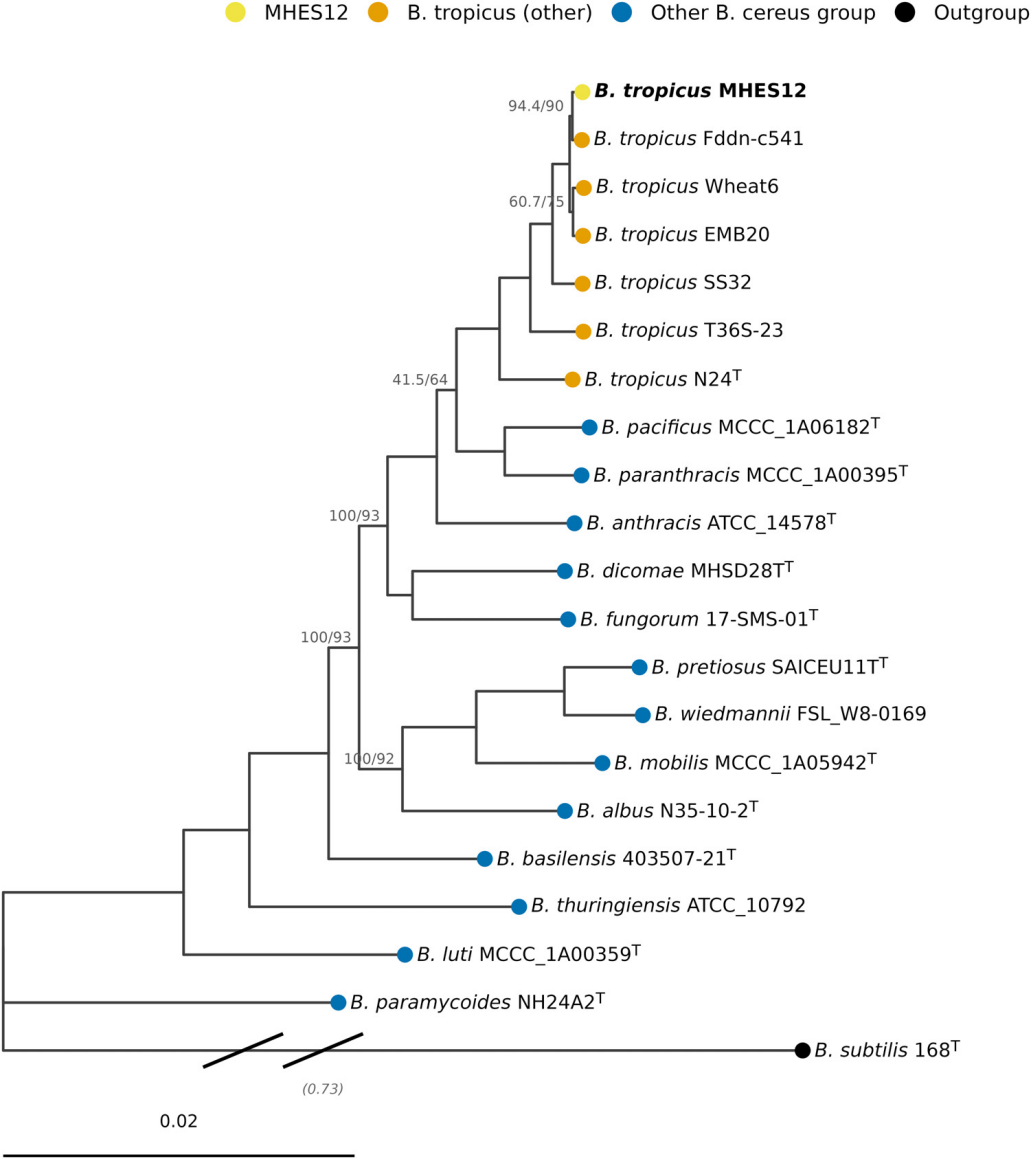
Maximum-likelihood core-genome phylogeny of *B. tropicus* MHES12 and 20 reference genomes [[7], [8], [9], [10], [11], [12], [13], [14], [15], [16], [17], [18], [19]], reconstructed from 1119 single-copy core genes (350,912 aligned amino-acid positions; LG+G4 model; IQ-TREE v3.1.2). Node values indicate SH-aLRT/ultrafast-bootstrap support (%) and are shown only where support falls below 80% SH-aLRT or 95% UFBoot; unlabelled internal nodes have 100/100 support. Superscript T denotes type strains. The tree is rooted on *Bacillus subtilis* 168 (outgroup branch length = 0.729 substitutions/site). MHES12 (bold) falls within a strongly supported (100/100) *B. tropicus* clade together with the type strain N24ᵀ and the publicly available *B. tropicus* EMB20, consistent with independently derived pairwise identity/relatedness values (FastANI, 96.98%; skani, 97.21%; NCBI ANI, 96.90%; TYGS dDDH d4, 72.5%). Scale bar, substitutions per site.

Taxonomic placement was verified by whole-genome average nucleotide identity and digital DNA-DNA hybridisation against 20 reference genomes (14 type strains of *B. cereus*-group species plus four in-house *B. tropicus* draft assemblies and one publicly available complete *B. tropicus* genome (EMB20); *B. subtilis* 168 as outgroup). All three ANI methods returned 96.9–97.2% identity between MHES12 and *B. tropicus* N24 (type strain, GCA_001884035.1) as the closest match, above the 95% species-delimitation threshold (FastANI 96.98%, skani 97.21%, NCBI ANI 96.90%) (Fig. 2, Table S8). TYGS reported dDDH d4 = 72.5% (CI 69.4–75.3%) to *B. tropicus* N24, above the 70% species threshold; all remaining subjects were below 70% in d4 (Figure S1). The TYGS-selected reference *Streptomyces microflavus* JCM 4496 exhibits an anomalous d4 value of 60.0%; however, its GC-content difference of 36.13% (versus ≤0.34% for all *Bacillus* subjects) indicates that the alignment covers only short conserved fragments and does not represent a valid inter-genome distance (Figure S1). A maximum-likelihood core-genome phylogeny built from 1119 single-copy core genes (350,912 aligned amino-acid positions; LG+G4 model; IQ-TREE v3.1.2; 1000 ultrafast bootstrap and 1000 SH-aLRT replicates) placed MHES12 inside a strongly supported (100% SH-aLRT/ultrafast-bootstrap support) *B. tropicus* clade together with the type strain N24, four in-house *B. tropicus* drafts (T36S-23, SS32, Wheat6, Fddn-c541), and the publicly available *B. tropicus* EMB20 (Fig. 3). Unlike the previously characterised *B. tropicus* EMB20, which was isolated from Indian soil and studied for β-lactamase activity [7,8], MHES12 originates from a drought-prone tomato rhizosphere in Bangladesh and carries a distinct stress-adaptation gene repertoire, providing complementary genomic context for the species.

## 4 Experimental Design, Materials and Methods

### 4.1 Strain isolation and identification

*B. tropicus* strain MHES12 was isolated from the rhizosphere of tomato plants grown in a drought-prone ecosystem in Rajshahi, Bangladesh (24.44,067° N, 88.40,943° E). Soil samples were collected aseptically, transported to the laboratory under cold-chain conditions, and processed within 12 h of collection. Isolation was performed by serial dilution and plating on yeast-extract peptone (YEP) agar, followed by incubation at 28 °C for 48–72 h. Presumptive colonies were purified by repeated streaking. Species identification was confirmed using the VITEK 2 Compact automated identification system (biomérieux) and *16S rRNA* gene PCR amplification and Sanger sequencing.

### 4.2 Genomic DNA extraction and quality assessment

Genomic DNA was extracted from an overnight culture of MHES12 using the GeneJET Genomic DNA Purification Kit (Thermo Fisher Scientific) following the manufacturer’s protocol. DNA purity (A260/A280 and A260/A230 ratios) and concentration were assessed using a NanoDrop 2000 spectrophotometer and confirmed using a Qubit 4.0 Fluorometer with the dsDNA BR Assay Kit (Thermo Fisher Scientific).

### 4.3 Library preparation and whole-genome sequencing

Sequencing libraries were prepared from 1 ng of input genomic DNA using the Nextera DNA Flex Library Preparation Kit (Illumina) according to the manufacturer’s instructions. Paired-end sequencing (2 × 150 bp) was performed on the Illumina MiSeq platform at 63.1× raw total-bp coverage (52.9× post-trim).

### 4.4 Quality control and genome assembly

Raw reads were assessed for quality using FastQC v0.11.3 [20]. Adapter trimming and quality filtering were performed using Trimmomatic v0.39 [21] with LEADING:3, TRAILING:3, SLIDINGWINDOW:4:20, MINLEN:50. Total read counts, Q20/Q30 percentages, GC content and duplication rates before and after trimming were computed with seqkit v2.5.1 [22] stats and the FastQC HTML reports (Table S1). High-quality reads were de novo assembled using SPAdes v4.2.0 [23] with default parameters. Assembly metrics were recomputed with QUAST v5.2.0 [24]. Genome completeness and contamination were re-evaluated using CheckM2 v1.0.2 [25] with the Neural-Network specific model against GTDB release Rr220; note that CheckM v1.2.5 [26] had been used in the initial submission. Sequencing coverage was calculated as the total number of sequenced bases divided by the assembled genome size (63.1× for raw reads and 52.9× after trimming); this bases-per-genome estimate is higher than the SPAdes-reported value (≈29×, Table 1), which reflects average k-mer coverage of the assembly graph rather than raw base coverage.

### 4.5 Genome annotation and functional analysis

Genome annotation was performed using the NCBI Prokaryotic Genome Annotation Pipeline (PGAP) v6.11 [27]. Functional subsystem analysis was conducted using the RAST server [28]; RAST subsystem annotations were used as reported by the server without manual curation, and functional assignments were cross-checked against Prokka and eggNOG-mapper outputs. Biosynthetic gene clusters (BGCs) were identified using antiSMASH v8.0 [29]. CRISPR arrays were detected using CRISPRCasFinder [30]. Plasmid replicons were screened using PlasmidFinder [31]. Human pathogenic potential was assessed using PathogenFinder2 [32]. Antimicrobial resistance and virulence-associated genes were screened using ABRicate v1.4.0 against CARD, ResFinder, NCBI-AMR, and VFDB databases with a minimum sequence identity threshold of ≥90% and minimum coverage of 90% (Table S3). Functional annotations were cross-checked with eggNOG-mapper [33] and Prokka [34].

### 4.6 Phylogenetic analysis and genome visualisation

Whole-genome average nucleotide identity between MHES12 and 20 reference genomes (14 *Bacillus cereus*-group type strains, four in-house *B. tropicus* drafts, one publicly available complete *B. tropicus* genome (EMB20), and *Bacillus subtilis* 168 as outgroup) was computed with FastANI v1.34 [35], skani v0.3.2 [36], and NCBI's web-hosted ANI Report tool [37]. Digital DNA-DNA hybridisation was computed with the Type Strain Genome Server (TYGS) using the Genome-BLAST Distance Phylogeny (GBDP) algorithm [38]. For core-genome phylogenomics, predicted proteomes of the 21 genomes (116,754 sequences) were clustered with MMseqs2 easy-cluster [39] at 50% amino-acid identity and 80% bidirectional coverage. The 1119 clusters containing exactly one member from each genome were used as the single-copy core. Each cluster was aligned with MAFFT v7.526 auto mode [40] and concatenated into a supermatrix of 350,912 aligned amino-acid positions. A maximum-likelihood tree was inferred with IQ-TREE v3.1.2 [41] under the LG+G4 model with 1000 ultrafast bootstrap and 1000 SH-aLRT replicates, rooted on *B. subtilis* 168. Trees were visualised with ggtree v3.14 [42] in R v4.4.2 [43], ANI matrices with ComplexHeatmap v2.22 [44], and dDDH bars with ggplot2 [45]. Strain metadata and accession numbers for all 21 genomes used in phylogenetic analyses are provided in Table S8 and the accompanying tree_strains_metadata file. The circular genome map was constructed using GenoVi [46].

## Limitations

The genome was assembled from short-read Illumina sequencing data alone, resulting in a draft assembly of 40 contigs rather than a closed circular genome. This fragmented assembly precludes resolution of long repeats, rRNA operon copy numbers, and any extrachromosomal replicons that may be present. All functional predictions reported here including metal-homeostasis, azo-dye degradation, osmotic- and oxidative-stress adaptation, and biosynthetic-gene-cluster products are based on sequence homology alone and have not been validated by targeted phenotypic or biochemical assays. The dataset represents a single isolate from one geographic location in Bangladesh and is not intended to be representative of broader *Bacillus tropicus* diversity. The presence of the *B. cereus* group enterotoxin operons (nheA/B/C, hblA/C/D, cytK) means that MHES12 should be considered for cytotoxicity testing before application.

## Funding

This research was supported by Research Management Wing, Gazipur Agricultural University (Project number: 002, Financial year: 2024–2027), Bangladesh.

## Ethics Statement

The authors have read and follow the ethical requirements for publication in Data in Brief and confirm that the current work does not involve human subjects, animal experiments, or any data collected from social media platforms. The study involved environmental soil sampling from agricultural land for research purposes, which did not require institutional ethical committee approval.

## CRediT Author Statement

**Md. Nazmul Hannan:** Conceptualization, Methodology, Investigation, Writing – Original Draft. **Md. Nayeem Hossain:** Methodology, Investigation, Formal Analysis, Writing – Original Draft. **Rifat Islam:** Investigation, Data Curation, Writing – Original Draft. **Sabbir Khan:** Software, Formal Analysis, Data Curation, Visualization, Writing – Original Draft. **Kamrun Nahar:** Methodology, Investigation, Resources. **GKM Mustafizur Rahman:** Conceptualization, Supervision, Writing – Review & Editing. **Habibul Bari Shozib:** Resources, Supervision. **Abul Hossain Molla:** Supervision, Writing – Review & Editing. **M. Nazmul Hoque:** Conceptualization, Supervision, Project Administration, Writing – Review & Editing. **Md. Manjurul Haque:** Conceptualization, Supervision, Project Administration, Writing – Review & Editing.

## Data Availability

NCBIBacillus tropicus strain MHES12, whole genome shotgun sequencing project (Original data).

## Appendix Supplementary materials

Image, application 1

Image, application 2

Image, application 3

Image, application 4

Image, application 5

Image, application 6

Image, application 7

Image, application 8

Image, application 9
