# Supplementary material for: Whole-genome sequence data of Bacillus tropicus strain MHES12 isolated from the rhizosphere of tomato in a drought-prone ecosystem in Bangladesh
Source: Data Brief. 2026 Aug 5;68:113123. doi: 10.1016/j.dib.2026.113123 (PMC13487590; doi:10.1016/j.dib.2026.113123)
Supplement: Supplementary file 1 [file mmc1.docx]

**Figure S1.** Digital DNA–DNA hybridisation (dDDH) values between *Bacillus tropicus* MHES12 and 15 reference genomes computed via TYGS (GBDP algorithm). Bars indicate dDDH percentages calculated under three distance formulas: d0 (HSP length/total length), d4 (identities/HSP length), and d6 (identities/total length). The dashed vertical line represents the 70% species-delimitation threshold. Under the d4 formula which is recommended for draft genomes only *B. tropicus* N24ᵀ crosses the threshold (72.5%), confirming conspecific with MHES12. While several non-target strains exceed 70% under the length-dependent d0 and d6 formulas, they are correctly delimited under d4. Notably, the TYGS-selected reference Streptomyces microflavus JCM 4496 exhibits an anomalous d4 value of 60.0; however, its GC-content difference is 36.13% (compared to ≤0.34% for all *Bacillus* subjects), indicating the alignment covers only short conserved fragments and does not represent a valid inter-genome distance.
